# Genome Sequence of *Pseudomonas* sp. Strain Chol1, a Model Organism for the Degradation of Bile Salts and Other Steroid Compounds

**DOI:** 10.1128/genomeA.00014-12

**Published:** 2013-01-15

**Authors:** Johannes Holert, Intikhab Alam, Michael Larsen, André Antunes, Vladimir B. Bajic, Ulrich Stingl, Bodo Philipp

**Affiliations:** a University of Münster, Institute of Molecular Microbiology and Biotechnology, Münster, Germany; c Red Sea Research Center; b Computational Bioscience Research Center, King Abdullah University of Science and Technology (KAUST), Thuwal, Saudi Arabia

## Abstract

Bacterial degradation of steroid compounds is of high ecological and biotechnological relevance. *Pseudomonas* sp. strain Chol1 is a model organism for studying the degradation of the steroid compound cholate. Its draft genome sequence is presented and reveals one gene cluster responsible for the metabolism of steroid compounds.

## GENOME ANNOUNCEMENT

Steroids are ubiquitous compounds with various functions in eukaryotic organisms. Bacteria from diverse phylogenetic groups are able to degrade steroid compounds. Distinct steps in bacterial steroid degradation are employed in biotechnology for the production of steroid drugs (1). In addition, bacterial degradation of hormonally active steroids, which may act as endocrine disrupters (2), is of ecological importance. Despite their relevance, the metabolic pathways for bacterial steroid degradation are still largely unexplored. The most information is available for cholesterol degradation by *Actinobacteria* (such as *Mycobacterium tuberculosis* and *Rhodococcus jostii*) (3), for testosterone degradation by the betaproteobacterium *Comamonas testosteroni* (4), and for cholate degradation by the gammaproteobacterium *Pseudomonas* sp. strain Chol1 (5). While genomes of steroid-degrading *Actinobacteria*, such as *Rhodococcus jostii* strain RHA-1 (6) and of two *C. testosteroni* strains (7, 8) are available, a genome sequence of a steroid-degrading gammaproteobacterium has not been published so far.

*Pseudomonas* sp. strain Chol1, a soil isolate, can grow with cholate and other steroid compounds as carbon and energy sources (9). Genomic DNA was extracted from cholate-grown cells of the strain Chol1 with a blood and cell culture DNA minikit (Qiagen).

Genome sequencing was performed using a combination of Roche 454 GS (FLX titanium) and Illumina (single and paired-end) sequencing platforms. A total of 74,399,617 bp (mean read length of 261 bp) was obtained from Roche 454, providing approximately 17-fold coverage. Single and paired-end sequences obtained by Illumina provided 124,765,470 bp (mean read length of 30 bp) and 287,012,740 bp (mean read length of 35 bp), respectively, corresponding to a 277-fold coverage. Sequences obtained with Roche 454 were assembled using a Newbler Assembler, version 2.5 (Roche), while sequences obtained by Illumina were assembled with SOAPdenovo (http://soap.genomics.org.cn/soapdenovo.html). Assemblies were merged using AMOS Minimus2 (http://sourceforge.net/apps/mediawiki/amos/index.php?title=Minimus2). The sequences were assembled into 42 scaffolds (N50 length 130 kb) from 199 contigs (N50 length 79 kb). N50 is the contig length, such that at least 50% of the bases of the assembly are contained within contigs of this length or greater. Genes were identified using Prodigal software (10) followed by mpiBLAST (http://www.mpiblast.org/) and EBI-Interproscan (http://www.ebi.ac.uk/) annotation matching data in public databases. This approach provided annotation for 93% of all 4,579 predicted genes. The draft genome has a G+C content of 63%.

The draft genome contains a 79-kb gene cluster (C211_11247—C211_11577) with obvious functions in steroid degradation including *acaD* (11) and *skt* (12), which were previously shown to be essential for cholate degradation. Genes within this cluster show higher similarity to homologs in *C. testosteroni* than to homologs in actinobacterial genomes, suggesting differences in the pathways for steroid degradation between Gram-positive and Gram-negative bacteria.

### Nucleotide sequence accession numbers.

This Whole Genome Shotgun project has been deposited at DDBJ/EMBL/GenBank under the accession number AMSL00000000. The version described in this paper is the first version, AMSL01000000.
